# Supplementary material for: A star-nose-like tactile-olfactory bionic sensing array for robust object recognition in non-visual environments
Source: Nat Commun. 2022 Jan 10;13:79. doi: 10.1038/s41467-021-27672-z (PMC8748716; doi:10.1038/s41467-021-27672-z)
Supplement: Supplementary file 1 — Supplementary information [file 41467_2021_27672_MOESM1_ESM.pdf]

SUPPLEMENTARY INFORMATION

A star-nose-like tactile-olfactory bionic sensing array for robust object recognition in non-visual environments

Mengwei Liu[1,2]†, Yujia Zhang[1,2]†, Jiachuang Wang[1,2]†, Nan Qin[1], Heng Yang[1,2], Ke Sun[1,2], Jie Hao[3], Lin Shu[3], Jiarui Liu[3], Qiang Chen[4], Pingping Zhang[5], Tiger H. Tao[1,2,6,7,8,9,10,11]*

Supplementary Notes:

1. Design and working principle of the force sensor.

Supplementary Figure 1 shows the magnified view of the force sensor along the single-crystalline silicon beam (blue area of the scanning electron microscope image in Figure 2a) and as shown in the image, the piezoresistive Wheatstone-bridge is integrated in the silicon beam. The thickness (5μm) of the silicon beam-island structure is designed as 2 to 3 times of the underneath poly-silicon diaphragm thickness (2μm) to achieve both high sensitivity and low nonlinearity. When an external force is applied to the force sensor, deflection of silicon membrane would be formed and then cause a change in the piezoresistive Wheatstone-bridge output value. Because of the design of the sensitive silicon beam and the integrated piezoresistive Wheatstone-bridge, the sensor is less affected by external conditions such as temperature and humidity change and can measure external force changes more accurately[1,2].

In detail, when the force sensor is in contact with the detected object, the membrane would form a certain deformation, the degree of which depends on both the stiffness of the object and the force applied. Then the membrane deformation would cause the changes in resistance values of the four piezoresistors. Thus, the voltage output value of the Wheatstone-bridge could reflect the change of resistance value and the degree of membrane deformation.

2. Detection of local topography by tactile sensing arrays.

As shown in Supplementary Figure 8 (a), height gradient with each step around 0.1 mm could be built by stacking various amount of A4 paper together; the single tactile sensing array (1*14) is used to perceive the existence of the paper. As shown in Figure R1 (b), the minimum detectable height difference for our tactile sensing array is around 0.3 mm, while larger height difference (0.3 to 1.5 mm) can be differentiated. Thus, the results of this experiment proves our tactile sensing array can perceive local topography with limit of detection of 0.3 mm.

First, as shown in Supplementary Figure 8 (a), the thickness of each force sensor is around 0.1 mm and every sensor is covered by soft silica gel to prevent them from damages caused in the touching process as mentioned in the experimental section. Thus, when one tactile sensor array (a row of 14 force sensors) is in contact with the surface of the detected object, not only the 14 sensors are in contact with the object, but the substrate (blue layer in Supplementary Figure 8 (a)) will also be in contact with the object at the same time. In this case, different contact surfaces will lead to different force distribution on the force sensors and the substrate below. Therefore, the 14 force sensors respond differently when the same overall force is applied across different step heights—we can only quantitatively apply an overall force on the whole sensor array—because of the variation of force dispersion on the substrate.

Furthermore, to prove the local topography of the more practical object can be perceived from a few sensors mounted at each fingertip alone, we used the tactile sensing arrays (5*14) mounted on the fingertips of a mechanical hand to detect the roughness of apple and orange surfaces. As shown in Supplementary Figure 9 (b) and (d), single tactile sensing array can accurately present the surface roughness differentiation between the tested objects. Thus, the local topography of tested objects can be obtained from the five tactile sensing arrays mounted at each fingertips of a mechanical hand (Supplementary Figure 9 (a) and (c)).

3. Detection of objects stiffness by tactile sensing arrays.

Supplementary Figure 2 (a) shows the sensitive hexagonal silicon membrane within the force sensor, on which located a Wheatstone bridge made by four piezoresistors. In principle, when the force sensor is in contact with the detected object, the membrane would form a certain deformation, the degree of which depends on both the stiffness of the object and the force applied. Then the membrane deformation would cause the changes in resistance values of the four piezoresistors. Thus, the voltage output value of the Wheatstone bridge could reflect the change of resistance value and the degree of membrane deformation.

Based on this working principle, when we use the mechanical hand to apply the same amount of force to touch the object with uniform topography, the output voltage value would be positively correlated to the local stiffness. As shown in Supplementary Figure 9 (b) and (d), because the stiffness of apple is relatively larger than orange, the average output voltage value of the sensor array for apple (around 5V) is larger than which for orange (around 2.5V). This result proves the capability of our tactile sensing arrays to distinguish the stiffness of various objects, providing important information for the following object recognition process.

Moreover, as shown in Figure 2 (d), F_{active} represents the average applied force and $F_{reactive}$ demonstrates the reactive force from the object to the sensor. When the same force is applied to different objects by the mechanical hand, the deformation degree of the objects varies according to objects' different elasticity moduli, which would change the local contact area and result in a different value of $F_{reactive}$. For example, when contacting an ideal rigid object with zero deformation, F_{active} is the same as $F_{reactive}$; when contacting a soft object with large deformation, the overall reactive force disperses both on the sensor ($F_{reactive}$) and the substrate, resulting in $F_{active} > F_{reactive}$.

In tactile perception, we mainly focused on the detection of the local topography (roughness) and material stiffness of the detected objects in order to distinguish human from other objects.

Compared to the traditional shape-based objects recognition through tactile perception, our method requires a smaller tactile sensing array. Moreover, in the design of the distribution of tactile sensors, we located the sensing arrays on the fingertips of the mechanical hand to gain information of local topography (roughness) and material stiffness. According to the limited area of each fingertip, 14 force sensors have been chosen in our design as a trade-off to form a single sensing array, which can acquire enough information for further objects recognition.

4. Design and working principle of the gas sensor.

In the design and fabrication of gas sensor, the platinum heating resistance wire is deposited on the insulating substrate to make sure the sensor works at an appropriate temperature, with a cavity below ensuring the concentration of heat. Then an insulating layer, made of silicon oxide and silicon nitride composite, is deposited by PECVD on top of the heating resistance. After that, the top layer is a pair of cross finger electrode covered with gas-sensitive material, the resistance change of which is an important criterion for gas detection. While the gas sensor is working under a specified temperature, the resistance of the cross finger electrode pair decreases as the gas molecules being adsorbed by the gas-sensitive materials. Thus, according to the degree of the resistance change, the gas sensor can measure both the types and concentration of the tested gas. Besides, different sensitive materials have various responses to specific gas molecules, thus by modifying gas sensors with different gas sensitive materials and forming a gas sensing array, the identification accuracy of gas type and concentration can be improved.

The working principle of the gas sensor is similar to that of the conventional semiconductor resistive sensor. While placing the sensors in the certain gas environment, the detected gas molecules would combine to the surface of the sensitive semiconductor material and a corresponding chemical reaction would occur. In this process, the electron transfer produced by

the chemical reaction could cause the resistance changes of the semiconductor material. By measuring the resistance change of the material, the status of the gas detection could be known. However, these reactions are not absolutely specific, which means instead of responding to a certain type of gas molecule, one sensor could respond to multiple gases in different responding degree. For example, the sensor number one would react to various gases, but only has the highest response to ethanol. Therefore, through multiple experiments, we can know the corresponding reaction sensitivity of these materials to different gas molecules. The gas types, including ethanol, acetone, ammonia, carbon monoxide, hydrogen sulfide and methane, as we mentioned in the article, are the most responsive gases corresponding to the six materials according to the experiment results. More importantly, since the sensitivities of each material to different gas molecules are different, we select the complementary setup (6 gas sensors) to sense the type and concentration of various gases, reflecting the odor profile of the detected object.

In olfactory perception, six gas sensors have been chosen for the detection of six specific gases, including ethanol, acetone, ammonia, carbon monoxide, hydrogen sulfide, and methane, which play an important role in object recognition. For example, ammonia and hydrogen sulfide are common components of biological odors, which could help to distinguish animal and human from other objects. Furthermore, by differentiating the concentration of these two gases, human can be distinguished from other animals such as mice (See confusion matrix from Figure 3c in the manuscript, page 31). Thus, based on this six-channel olfactory sensing array, efficient odor recognition of different objects can be achieved.

5. Olfactory and tactile perception of oranges being covered with liquids like water and muddy water.

As shown in Supplementary Figure 10, we use oranges (normal, covered with water and muddy water) as the target objects for identification. During this process, because of the encapsulation, the olfactory sensing array has a certain capability to withstand water environment, which is sufficient to detect objects covered with water or muddy water without causing any damage to sensors. However, for long-term underwater immersion (⩾1 day), our current sensor packaging method might not be sufficient and more improvements on this problem can be carried on in our future research.

Compared to the dry condition, when the orange is covered with water, the response degree of each olfactory sensor has been reduced slightly, because the increased humidity affects the responsive reaction. For the situation when orange is covered with muddy water, with the interference of the mud odor, the responses are further affected. However, while combining olfactory sensing with tactile perception, the interference of olfactory perception can be alleviated and a relative high recognition accuracy can be maintained whether the target object is cover with water or muddy water, which also demonstrates the outstanding anti-interference ability of the tactile-olfactory sensing system in our work.

6. Data processing

Due to device variation, a normalization step to preprocess the signals measured by tactile and olfactory sensing arrays is required before implementing machine learning. We smooth the output values first by Savitzky Golay filter and then rescale the rest dataset to [0,1] by using min-max normalization.

Each force sensor is individually calibrated. Firstly, the baseline (U_{max} and U_{min}) of the useful signals of the force sensor is determined. Here, U_{max} is the median value of multiple measurements when the detected object is fully touched by the mechanical hand, and U_{min} is the

median value of multiple measurements before touching. The min-max normalization of the tactile signals can be obtained by using the following formula:

$$U_{normalized} = \frac{U' - U_{min}}{U_{max} - U_{min}} \tag{1}$$

where U' represents the measured value of the force sensor after smoothing. A similar procedure is followed with the olfactory signals in normalizing the dataset by using the following formula:

$$R_{normalized} = \frac{R'}{R_0} \tag{2}$$

where R' represents the measured resistance value of the olfactory sensor after smoothing and R_0 is the initial resistance value of the olfactory sensor.

In addition to using the fundamental sampling method, we consider another two different strategies for tactile signal selection (one sample contains N tactile signals, N>1, aims to accommodate information from different grasp configurations and provide more varied sensory data). The first one is a simple random choice of N signals from the recording (This is used in the BOT-R). The second is aimed at maximizing the variance between the N signals. For this, we use principal component analysis to reduce the dimensionality of the tactile signals and find N clusters via k-means clustering. We then select N input signals from each cluster randomly (this is used in the BOT-F and BOT-M).

We also augment our training data by using several image processing techniques (rotating, cropping, translating and flipping) to simulate different touch gestures and reply various actual test data. Ensuring the consistency of sample number and the convenience of verification, we sample 5,000 samples for every type of object from above normalized dataset. In total, our tactile-olfactory (TO) dataset contains 55,000 TO samples. The data throughput we used is smaller than the other classification methods relying on vision or tactile[3-6]. Finally, we split the dataset into training and

test subsets, out of the five sets of trials in the TO dataset, four sets of trials for training, and one set of trials for testing.

7. Network design

We use a modified version of the VGG architecture as the base of our network (Supplementary Figure 9a)[7,8]. Following the same principle, the tactile and olfactory information collected by our sensors is sorted into parallel tactile image and olfactory sequence after data preprocessing. We analyze the feature vectors extracted by CNN, fuse them by MCB, and then use a fully connected neural network to complete the classification. Therefore, the CNN provides key features for the following algorithm, as an important and effective method for information processing. Because of the scale and characteristic of tactile data, we remove the most convolution layer and reduce the filter size of the max pooling. We also replace the fully connected layer that has 4096 neurons with 512 neurons. The new classification layer is of 11 categories instead of 1000. To reduce overfitting to our training set, we introduce the spatial dropout layers with 50% drop probability between the fully connected layers. For olfactory data, we design a fully connected neural network (Supplementary Figure 7b).

Firstly, we pertain the network on the tactile dataset and olfactory dataset separately. We truncate the final classification layer of the above two networks, and then fuse the learned tactile output (512D) and the learned olfactory representation (512D) by using Multimodal Compact Bilinear Pooling (MCB) to form a new vector (512D)[7,8]. After MCB pooling, a fully connected neural network connects the resulting multimodal representation to the classification.

One key point of the above network is the fusion. Approaches to multimodal pooling include element-wise product or sum, as well as concatenation[9]. Obviously, these methods are not expressive in dealing with complex tasks and do not reflect the basis of integration. We borrow

the MCB for the task of visual question answering (VQA) to efficiently and expressively combine multimodal features. It could realize an outer product of two vectors and reduce the amount of calculation. We prove that the performance of this fusion method is satisfactory in handling our dataset by some experiments.

Human always identify the object accurately by touching several times. Inspired by this, we propose the multiple decision algorithm (Supplementary Figure 9c). Briefly, when the result of the classification neural network is ambiguous, our system will cumulate the results and resample a new input, and then repeat the neural network algorithm until output and cycle number follow the certain relation. Overall, our multiple decision method is functional and improves the classification accuracy effectively. Additionally, in order to handle different situations, we also propose a scenario dependent feedback mechanism. This function is mainly accomplished by adjusting the parameters and method of fusion algorithm.

Finally, we implement the network in the PyTorch deep learning framework. We use Adam solver implemented in PyTorch to train our model and minimized the cross-entropy loss. We apply a learning rate of 0.001. We report the average results over 10 training runs at least. Similar methods are used for the training and classification tests used to evaluate in the other actual situations.

8. The bioinspiration and potential impacts of our sensing arrays:

Converging tactile and olfactory information in perceptual decision-making could improve the recognition accuracy compared to arbitrary single input. Meanwhile, the calculation-saving tactile-olfactory perception, compared to visual perception for instance, assists the mole to make nearly instantaneous judgment about the type of the touched objects as well as the following actions of the mole itself. Inspired by this characteristic of the star-nosed mole, recognition of

objects in various challenging environments can be achieved by integrating both tactile and olfactory sensing arrays on a mechanical hand with a combination of a BOT associated machine-learning architecture. Furthermore, the multisensory fusion strategy provides robust performance and anti-inference capability in contrast with unisensory perception under multiple hazards. Our work is promising for recognition tasks in rescue, industrial production and medical treatment applications, and inspiring for future biomimicking engineering, advanced sensor fabrication and integration, as well as machine learning[10-12].

Supplementary Figures:

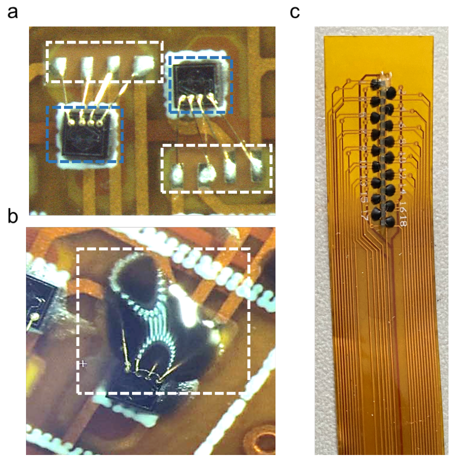

Supplementary Figure 1. Images of force sensors integrated on flexible PCB board. a) Photo of force sensors integrated on flexible PCB board after wire bonding. The blue dotted squares indicate the position of force sensors and the white dotted squares indicate the location of silver paste at the contact point. b) Photo of force sensors integrated on flexible PCB board with vinyl protection. The white dotted squares indicate the position of vinyl. c) Photo of force sensors integrated on flexible PCB board.

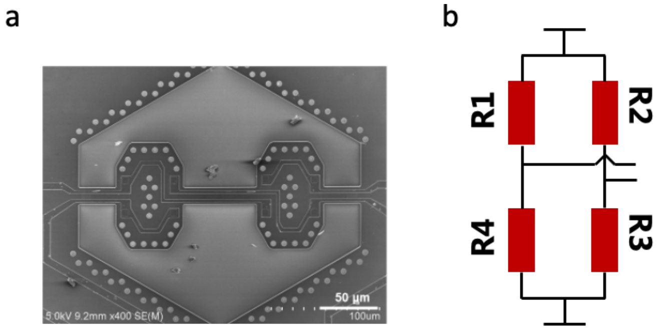

Supplementary Figure 2. Interior structure of a single force sensor. a) SEM image showing the sensitive hexagonal silicon membrane within the force sensor, on which located a Wheatstone bridge made by four piezoresistors. b) Schematic illustration of the electronic circuit of the four piezoresistors.

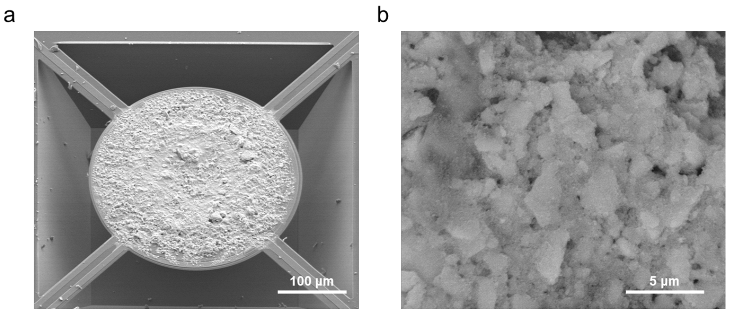

Supplementary Figure 3. Scanning electron microscope (SEM) images of gas sensor. a) SEM image of magnified view of the part modified by gas-sensitive material. b) SEM image of gas-sensitive material.

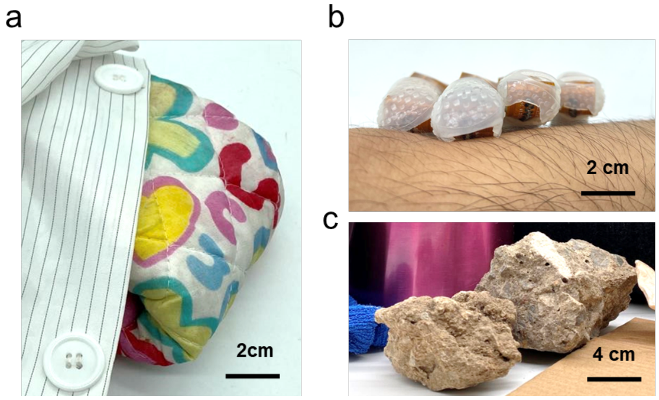

Supplementary Figure 4. Recognition of objects with various elastic stiffness based on force sensor. Photos of three testing objects including clothes (a), skin (b) and stone (c).

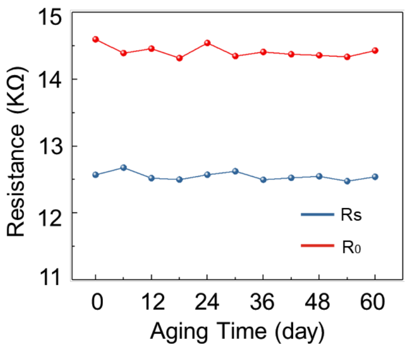

Supplementary Figure 5. Stable performance of gas sensor in air (red line) and in 100 ppm ethanol (blue line).

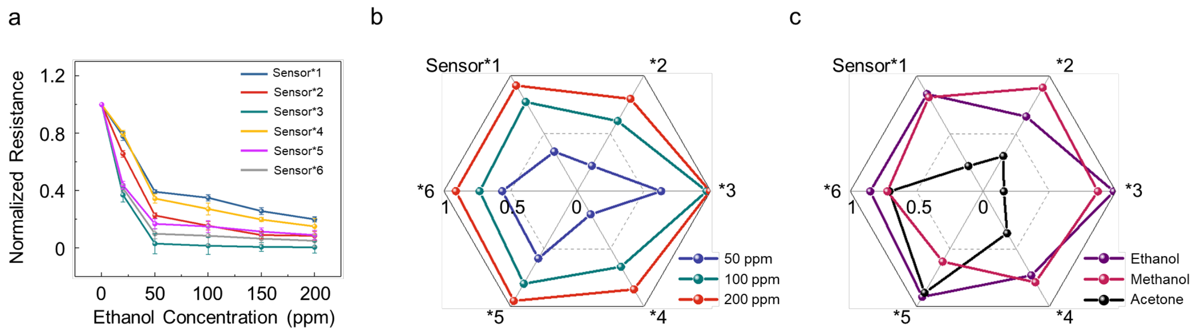

Supplementary Figure 6. Characterizations of six gas sensors. (a) Normalized resistance response curve of the 6 gas sensors in various ethanol concentration (50ppm, 100ppm, 150ppm and 200ppm). n = 12 for each group. The error bars denote standard deviations of the mean. (b) The response of six gas sensors in different gas concentration (c) The response of six gas sensors in different gas types.

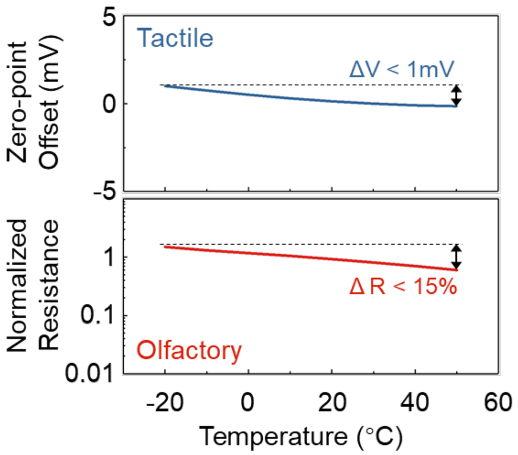

Supplementary Figure 7. The temperature stability of force (top) and gas sensors (bottom).

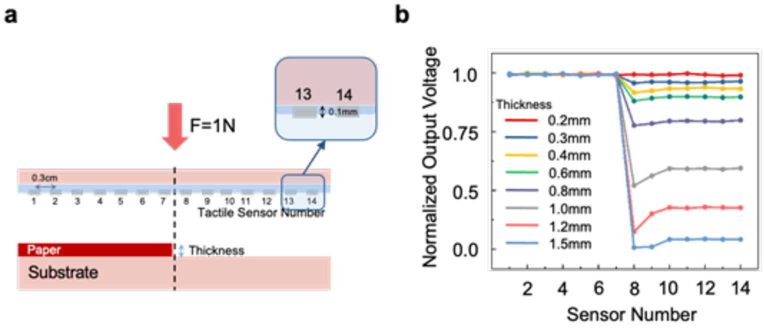

Supplementary Figure 8. Tactile perception of height gradient. a) Schematic illustration of using single tactile sensing array (1*14) to detect height gradient. b) Output voltage of force sensors while touching paper pile with thickness from 0.2mm to 1.5mm.

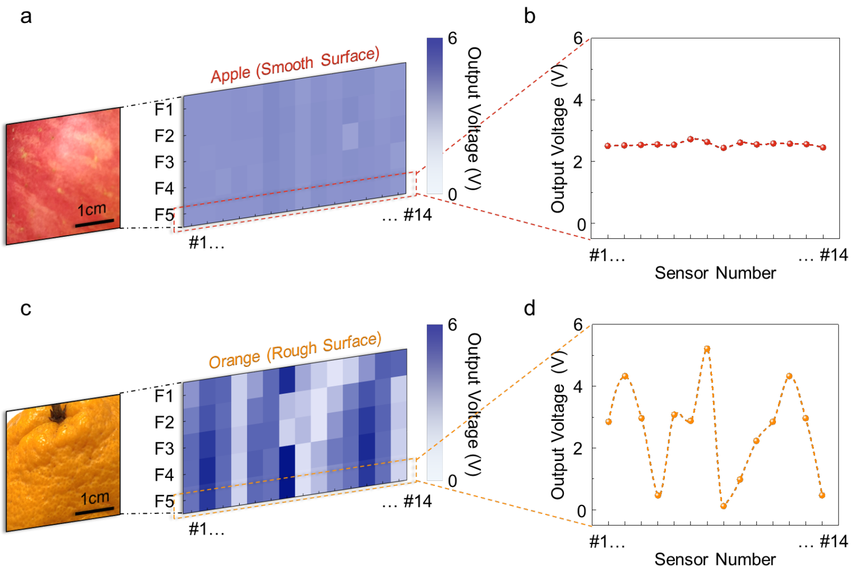

Supplementary Figure 9. Tactile perception of different objects with various surface roughness. a) The tactile mapping of the local topography of an apple surface, detected by the tactile sensing arrays (5*14) mounted on the fingertips of a mechanical hand. b) Output voltage of every force sensor from a single tactile sensing array (1*14), which present the local roughness of the apple surface. c) The tactile mapping of the local topography of an orange surface, detected by the same tactile sensing arrays (5*14). d) Output voltage of every force sensor from a single tactile sensing array (1*14), which present the local roughness of the orange surface.

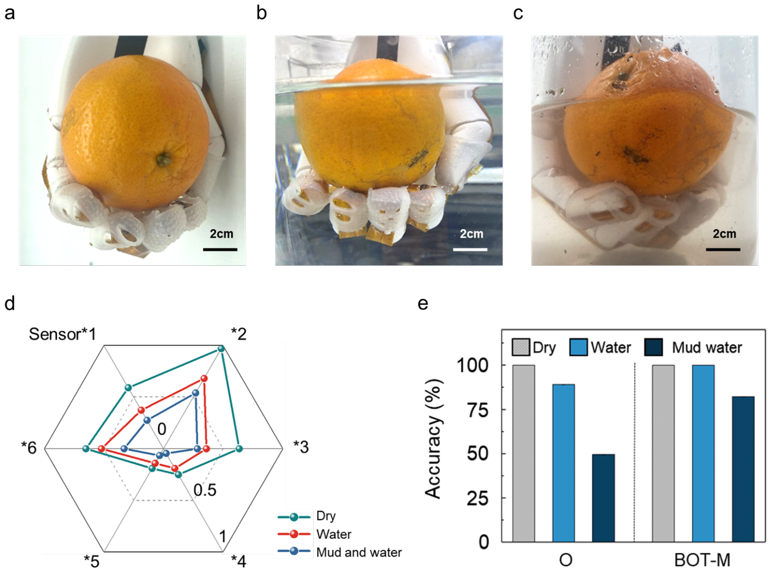

Supplementary Figure 10. Olfactory and tactile perception of oranges being covered with water and muddy water. a-c) Images of olfactory and tactile perception of orange in normal, covered with water, and covered with muddy water. d) The responses of six gas sensors when sensing orange covered with water and muddy water. e) The recognition accuracy of orange covered with water and muddy water using olfactory-based recognition and BOT-M associated learning.

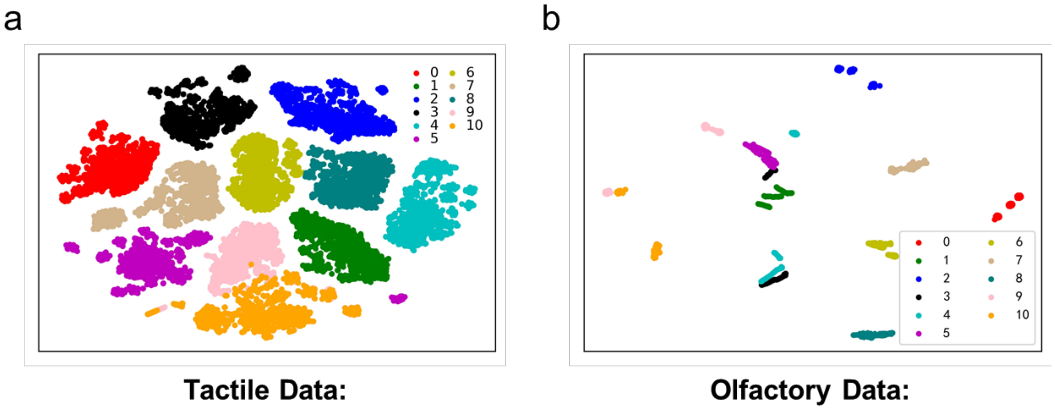

Supplementary Figure 11. The Visualizing of both tactile (a) and olfactory (b) information in the dataset using t-distributed stochastic neighbor embedding dimensionality reduction.

Each point represents tactile or olfactory information of one object recognition projected from the 70D tactile data or 6D olfactory data into two dimensions. Results of each object cluster together.

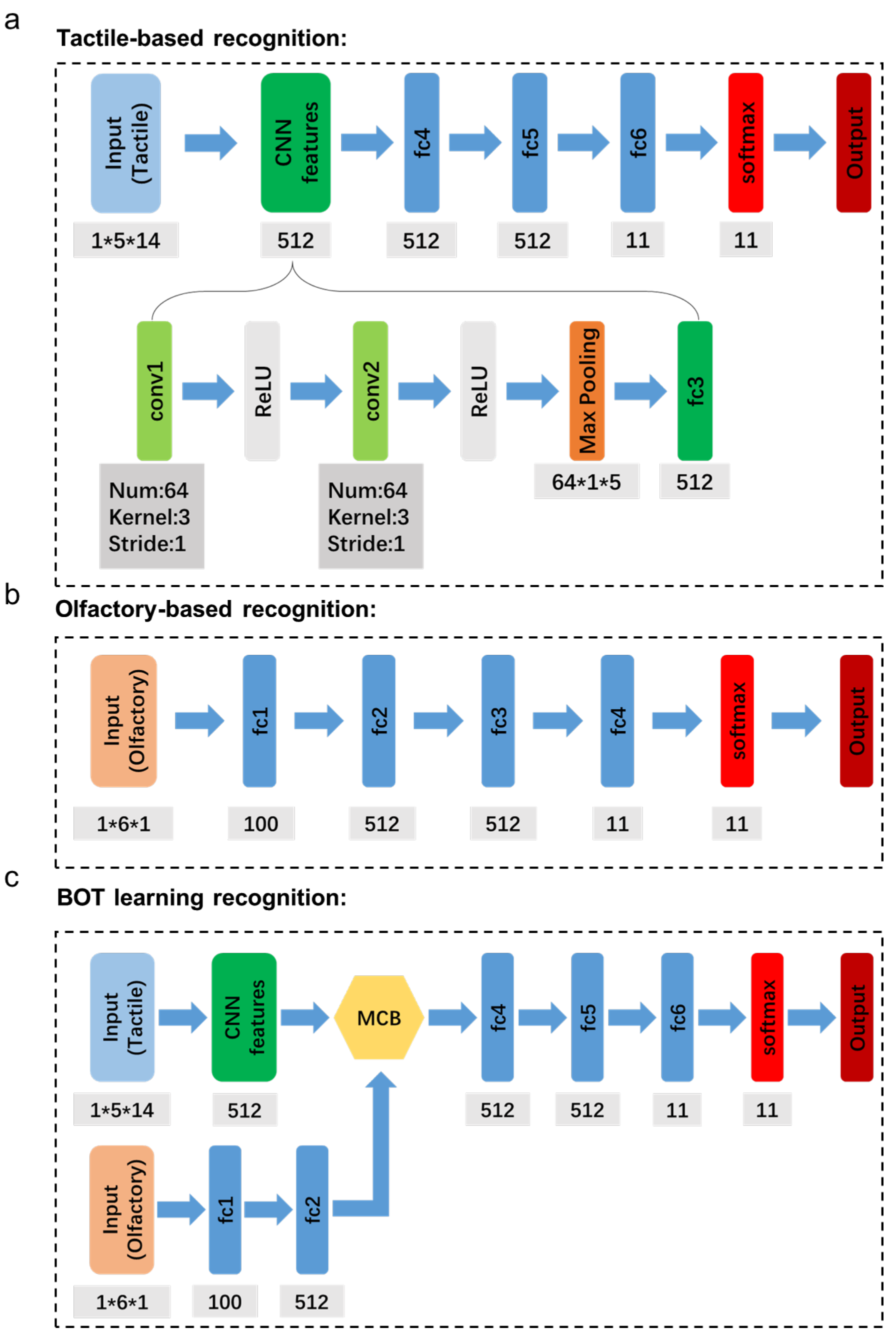

Supplementary Figure 12. Architectures of three recognition strategies. Unimodal strategies: tactile-based recognition only using force sensor data (a), and olfactory-based recognition only using gas sensor data (b). BOT associated learning recognition strategy using both force sensor

data and gas sensor data (c). CNN for tactile-based recognition, a 6-layer feedforward neural network for olfactory-based recognition. fc: fully connected neural network.

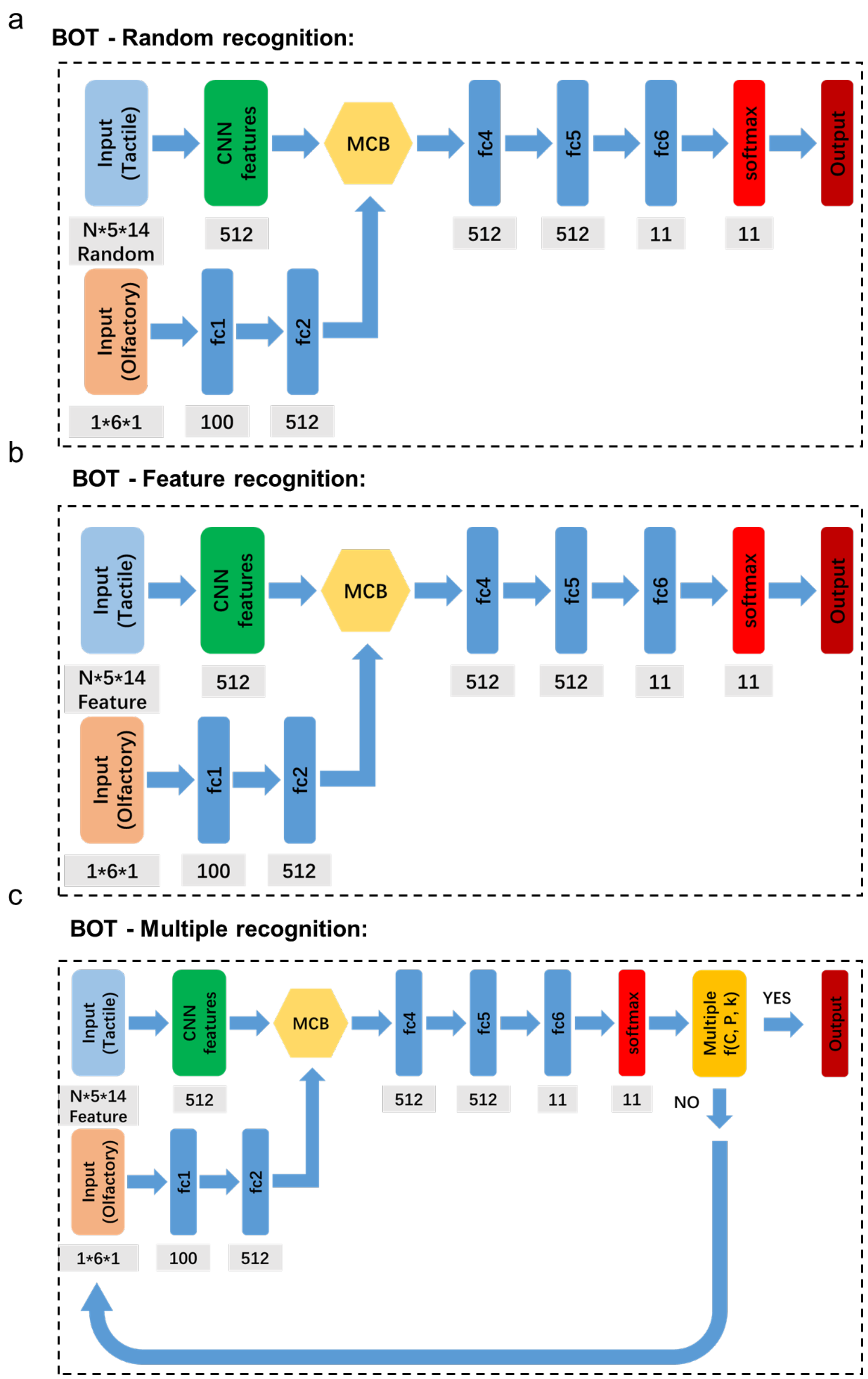

Supplementary Figure 13. Architectures of three tactile and olfactory fusion recognition strategies: BOT-R (a), BOT-R (b), BOT-M (c) using both force and gas sensing data.

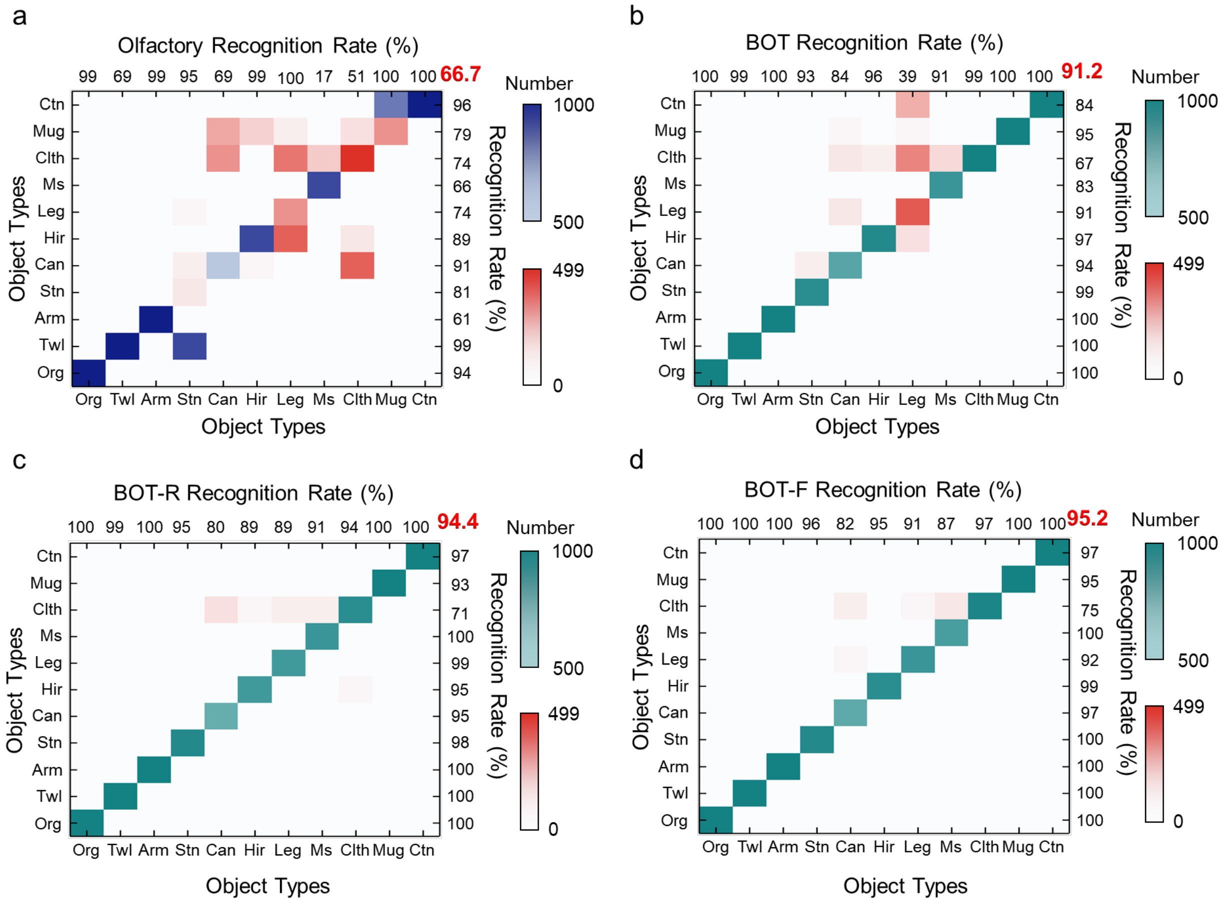

Supplementary Figure 14. Confusion matrices based on 4 different recognition strategies: olfactory, BOT, BOT-R and BOT-F. The full name of the abbreviation are: Org-Orange; Twl-Towel; Arm-Arm; Stn-Stone; Can-Can; Hir-Hair; Leg-Leg; Ms-Mouse; Clth-Worn Clothes; Mug-Mug; Ctn-Carton.

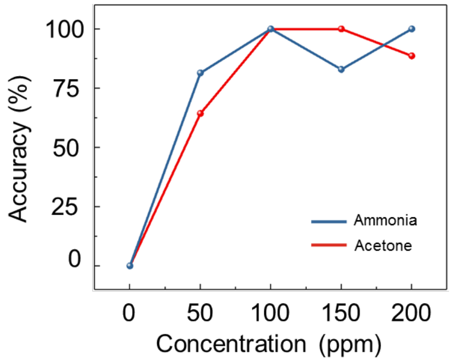

Supplementary Figure 15. Accuracy of identifying ammonia and acetone with various gas interference.

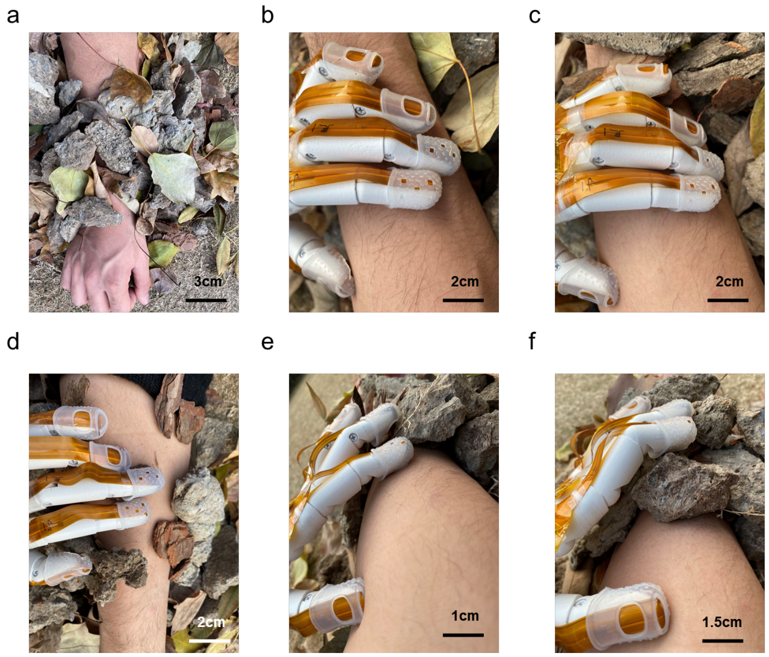

Supplementary Figure 16. Photos of arm recognition in the debris with various burial degree. Photos of none finger (b), one finger (c), two fingers (d), three fingers (e) and four fingers (f) of mechanical hand being blocked from touching the arm.

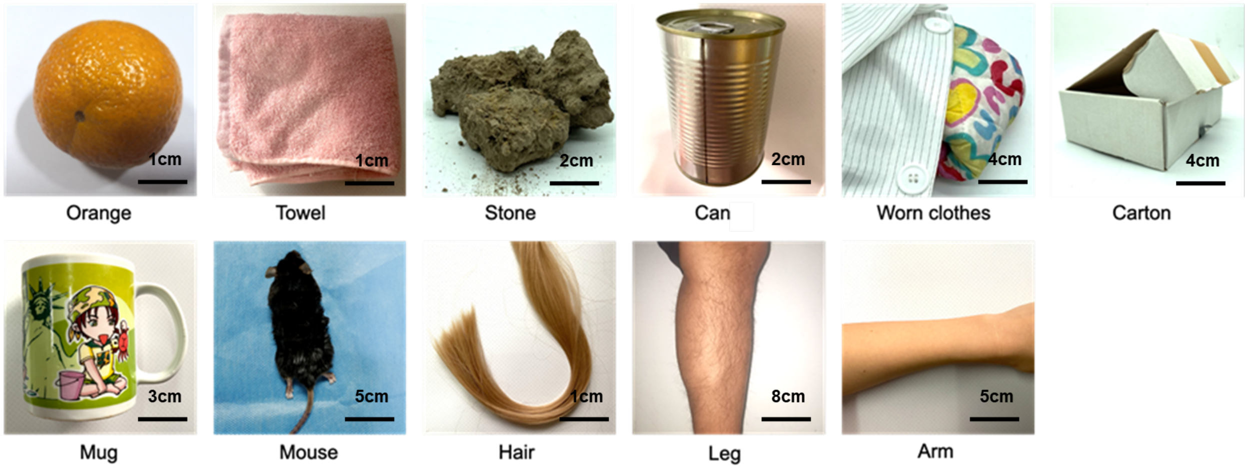

Supplementary Figure 17. Target objects for building the dataset. In total, 11 objects are used in our dataset, whose images are shown here.

Supplementary Tables:

Supplementary Table 1. The algorithm flow table showing the principle of designing our BOT architecture.

Algorithm 1: Multiple Classification Algorithm

Input: tactile dataset T, olfactory dataset O
Output: class C
initializtion
scenario dependant feedback n_t, n_o, d
while $\mathbf{P} \leq \overline{P}$ *and* $k \leq N$ **do**
    $k \leftarrow k + 1$
    $T_s, O_s \leftarrow Sample(T, O)$
    $T_{net} \leftarrow Net_T(T_s)$
    $O_{net} \leftarrow Net_O(O_s)$
    $I_{fusion} \leftarrow MCB(T_{net}, O_{net}, n_t, n_o, d)$
    $C, P \leftarrow Net_f(I_{fusion})$
    $\mathbf{C}_k \leftarrow C$
    $\mathbf{P} \leftarrow a_k \times \mathbf{P} + b_k \times P$
    if $k \geq N$ **then**
        $C \leftarrow Mode(\mathbf{C})$
    end
end

Supplementary Table 2. Comparison of different object recognition methods.

	Sensor type	Algorithm	Input size	Data size	Computing resource	Method	Multi-modality	Accuracy	Specialty	Deficiency	Ref.
**Need Imaging**	Depth camera	Dex-Net4.0	—	Large	Large	Depth image	No	95%	—	Need direct and clear visualization	[14]
	GelSight & RGB camera	ResNet50	128×128×3+ 256×256×3	Medium	Medium	Grasp & image	Yes	—	—	Need direct and clear visualization & unsuitable for varied grasping	[15]
	Stretchable strain & camera	Alex-Net	160×120+5	Light	Medium	Somatosensory & image	Yes	100%	Improved accuracy under dim light	Need large data size and computing resource	[4]
**Without Imaging**	GelSight	ResNet50	—	Medium	Medium	Dig(touch)	No	99%	Recognizing object submerged in sand	Need specific object & deficient anti-interference	[16]
	Tactile	ResNet18	32×32	Medium	Medium	Grasp	No	>90%	Learning the grasping pattern	Need manual grasping	[3]
	Haptic stimulator	CNN, SVM	16×200	Medium	Light	Grasp	Yes	96%	Providing haptic-feedback for human-machine interface	Need specific object & deficient anti-interference	[17]
	Quadruple tactile	MLP	4×10	Light	Light	Grasp	Yes	94%	Suitable for robotic hand	Limited workable environment & deficient anti-interference	[18]
	Pressure&vibration	ANN	—	Medium	Light	Touch	Yes	99.1%	Recognizing surface texture	Need specific object & limited algorithm	[19]
	**Tactile & olfactory**	**BOT (CNN/FCN/ MCB)**	**5×14+6**	**Light**	**Light**	**Touch**	**Yes**	**96.9%**	**Improved anti-interference & Suitable for rescue scenarios**	**Fully underwater environment**	**This work**

Supplementary Table 3. Comparison of different recognition patterns of animals in the dark environment.

Animal name	Class	Special Sensing	Biological organs	Suitable scenario	Reference
Bats	Mammal	Ultrasound	Throat/Ears	Location orientation	[20]
Octopus	Reptile	Light/Tactile	Skin	Camouflage/Prey	[21]
Snakes	Cephalopod	Infrared Radiation	Infrared receptor	Prey	[22,23]
Owl	Aves	Light/Sound	Eyes/Ears	Prey	[24]
Platypus	Mammal	Electroreception	Mucous glands	Prey/Underwater orientation	[25]
Catfish	Pisces	Taste	Whiskers	Prey	[26]
Cavefish	Pisces	Sound	Lateral line system	Prey	[27]
Mantis Shrimp	Malacostraca	Visible/ultraviolet light	Visual System	Prey	[38]
**Star-nosed mole**	**Mammal**	**Tactile/Olfactory**	**Tentacles/nostrils**	**Prey/Objects recognition**	**[29-30]**

Supplementary References

1. Wang, J. & Li, X. Single-Side Fabricated Pressure Sensors for IC-Foundry-Compatible, High-Yield, and Low-Cost Volume Production. *IEEE Electron Device Lett.* **32**, 979-981 (2011).

2. Wang, J. & Li, X. Package-friendly piezoresistive pressure sensors with on-chip integrated packaging-stress-suppressed suspension (PS3) technology. *J. Micromech. Microeng.* **23**, 045027 (2013).

3. Sundaram, S. *et al.* Learning the signatures of the human grasp using a scalable tactile glove. *Nature* **569**, 698-702 (2019).

4. Wang, M. *et al.* Gesture recognition using a bioinspired learning architecture that integrates visual data with somatosensory data from stretchable sensors. *Nat. Electron.* **3**, 563-570 (2020).

5. Yousef, H., Boukallel, M. & Althoefer, K. Tactile sensing for dexterous in-hand manipulation in robotics—A review. *Sensor Actuat. A-Phys.* **167**, 171-187 (2011).

6. Ficuciello, F., Migliozzi, A., Laudante, G., Falco, P. & Siciliano, B. Vision-based grasp learning of an anthropomorphic hand-arm system in a synergy-based control framework. *Sci. Robot.* **4**, eaao4900 (2019).

7. LeCun, Y., Bengio, Y. & Hinton, G. Deep learning. *Nature* **521**, 436-444 (2015).

8. Rusk, N. Deep learning. *Nat. Methods* **13**, 35-35 (2016).

9. Chen, A. et al. Multi-information fusion neural networks for arrhythmia automatic detection. *Comput. Methods Programs Biomed.* **193**, 105479 (2020).

10. Zhang, Y. & Tao, T. H. Skin-Friendly Electronics for Acquiring Human Physiological Signatures. *Adv. Mater.* **31**, 1905767 (2019).

11. Zhang, Y. & Tao, T. H. A Bioinspired Wireless Epidermal Photoreceptor for Artificial Skin Vision. *Adv. Funct. Mater.* **30**, 2000381 (2020).

12. Liu, M. *et al.* Biomimicking Antibacterial Opto-Electro Sensing Sutures Made of Regenerated Silk Proteins. *Adv. Mater.* **33**, 2004733 (2020).

13. Wang, G. F., Li, Y. S., Cai, Z. Z., Dou, X. C., A Colorimetric Artificial Olfactory System for Airborne Improvised Explosive Identification. *Adv. Mater.* **32**, 1907043 (2020).

14. Mahler J. et al. Learning ambidextrous robot grasping policies. *Sci. Robot.* **4**, 26 (2019).

15. Lin J, Calandra R, Levine S. Learning to Identify Object Instances by Touch: Tactile Recognition via Multimodal Matching. *International Conference on Robotics and Automation*.

16. Patel R, Ouyang R, Romero B, et al. Digger Finger: GelSight Tactile Sensor for Object Identification Inside Granular Media. 2102.10230v1 (2021).

17. Zhu M. et al. Haptic-feedback smart glove as a creative human-machine interface (HMI) for virtual/augmented reality applications. *Sci. Adv.,* 2020, 6(19).

18. Li G. et al. Skin-inspired quadruple tactile sensors integrated on a robot hand enable object recognition. *Sci. Robot,* 2020, 5(49):eabc8134.

19. Chun S. et al. An artificial neural tactile sensing system. *Nat. Electron*, 2021.

20. Fenton, Brock M . Questions, ideas and tools: lessons from bat echolocation. *Anim Behav*,

2013, 85(5):869-879.

21. Jinping, Hou, and, et al. Design of a Biomimetic Skin for an Octopus-Inspired Robot – Part I: Characterising Octopus Skin. *J Bionic Eng*, 2011.

22. Panzano V C , Kang K , Garrity P A . Infrared snake eyes: TRPA1 and the thermal sensitivity of the snake pit organ. *Sci Signal*, 2010, 3(127):pe22.

23. Molenaar G J . The sensory trigeminal system of a snake in the possession of infrared receptors. I. The sensory trigeminal nuclei. *J Comp Neurol*, 2010, 179(1):137-151.

24. Golubeva T V . Acoustically guided behavior in the early ontogeny of the long-eared owl: the development of hearing sensitivity. Zh Vyssh Nerv Deiat Im I P Pavlova, 1996, 46(2):317-327.

25. T Fjällbrant, Manger P R , Pettigrew J D . Some related aspects of platypus electroreception: temporal integration behaviour, electroreceptive thresholds and directionality of the bill acting as an antenna. *Phil. Trans. R. Soc. Lond*. B3531211–1219.

26. Caprio, John. High sensitivity of catfish taste receptors to amino acids. *Comp Biochem Physiol A Physiol*, 1975, 52(1):247-251.

27. Kulpa M , Bak-Coleman J , Coombs S . The lateral line is necessary for blind cavefish rheotaxis in non-uniform flow. *J Exp Biol*, 2015, 218(10):1603.

28. Zhang X , Jin W , Su Q . Researching Summary of the Mantis Shrimp's Visual Imaging Characteristics and Bionic Technology. *Infrared Techn*, (2016).

29. Wang, Z. et al. Star-nose-inspired multi-mode sensor for anisotropic motion monitoring. *Nano Energy,* 80, 105559 (2021).

30. Catania, K. C. Early development of a somatosensory fovea: a head start in the cortical space race? *Nat. Neurosci*. 4, 353-354 (2001).
