# Supplementary material for: A star-nose-like tactile-olfactory bionic sensing array for robust object recognition in non-visual environments
Source: Nat Commun. 2022 Jan 10;13:79. doi: 10.1038/s41467-021-27672-z (PMC8748716; doi:10.1038/s41467-021-27672-z)
Supplement: Supplementary file 3 — Description of Additional Supplementary Files [file 41467_2021_27672_MOESM3_ESM.docx]

**Description of Additional Supplementary Files**

**Title: Supplementary Movie 1.**

**Description:** Robotic arm performing rescues

**Title: Supplementary Movie 2.**

**Description:** Software user interface.
