# Supplementary material for: A star-nose-like tactile-olfactory bionic sensing array for robust object recognition in non-visual environments
Source: Nat Commun. 2022 Jan 10;13:79. doi: 10.1038/s41467-021-27672-z (PMC8748716; doi:10.1038/s41467-021-27672-z)
Supplement: Supplementary file 6 — Reporting Summary [file 41467_2021_27672_MOESM6_ESM.pdf]

nature portfolio

Corresponding author(s): Tiger H. Tao
Last updated by author(s): Nov 21, 2021

Reporting Summary

Nature Portfolio wishes to improve the reproducibility of the work that we publish. This form provides structure for consistency and transparency in reporting. For further information on Nature Portfolio policies, see our Editorial Policies and the Editorial Policy Checklist.

Statistics

For all statistical analyses, confirm that the following items are present in the figure legend, table legend, main text, or Methods section.

n/a	Confirmed	
☐	☒	The exact sample size (*n*) for each experimental group/condition, given as a discrete number and unit of measurement
☐	☒	A statement on whether measurements were taken from distinct samples or whether the same sample was measured repeatedly
☒	☐	The statistical test(s) used AND whether they are one- or two-sided<br>*Only common tests should be described solely by name; describe more complex techniques in the Methods section.*
☒	☐	A description of all covariates tested
☒	☐	A description of any assumptions or corrections, such as tests of normality and adjustment for multiple comparisons
☐	☒	A full description of the statistical parameters including central tendency (e.g. means) or other basic estimates (e.g. regression coefficient) AND variation (e.g. standard deviation) or associated estimates of uncertainty (e.g. confidence intervals)
☒	☐	For null hypothesis testing, the test statistic (e.g. *F*, *t*, *r*) with confidence intervals, effect sizes, degrees of freedom and *P* value noted<br>*Give P values as exact values whenever suitable.*
☒	☐	For Bayesian analysis, information on the choice of priors and Markov chain Monte Carlo settings
☒	☐	For hierarchical and complex designs, identification of the appropriate level for tests and full reporting of outcomes
☒	☐	Estimates of effect sizes (e.g. Cohen's *d*, Pearson's *r*), indicating how they were calculated

Our web collection on statistics for biologists contains articles on many of the points above.

Software and code

Policy information about availability of computer code

Data collection	Data collection listed in Methods. All software used in this study for data collection are either commercially available or open source. The Replication code that supports the plots within this paper and other findings of this study is available from GitHub at https://github.com/wjcbob/BOT.<br>DOI identifier: 10.5281/zenodo.5714516. year : 2021.
Data analysis	All software used in this study for data analysis are either commercially available or open source. The main software used for data analyzing includes Matlab R2020a and Origin 8.

For manuscripts utilizing custom algorithms or software that are central to the research but not yet described in published literature, software must be made available to editors and reviewers. We strongly encourage code deposition in a community repository (e.g. GitHub). See the Nature Portfolio guidelines for submitting code & software for further information.

Data

Policy information about availability of data

All manuscripts must include a data availability statement. This statement should provide the following information, where applicable:

- Accession codes, unique identifiers, or web links for publicly available datasets
- A description of any restrictions on data availability
- For clinical datasets or third party data, please ensure that the statement adheres to our policy

All data needed to evaluate the conclusions in the paper are present in the paper and the Supplementary information. Additional data related to this paper may be requested from the authors. The computational data is available from GitHub at https://github.com/wjcbob/BOT. DOI identifier: 10.5281/zenodo.5714516. year : 2021

Field-specific reporting

Please select the one below that is the best fit for your research. If you are not sure, read the appropriate sections before making your selection.

☐ Life sciences ☒ Behavioural & social sciences ☐ Ecological, evolutionary & environmental sciences

For a reference copy of the document with all sections, see nature.com/documents/nr-reporting-summary-flat.pdf

Behavioural & social sciences study design

All studies must disclose on these points even when the disclosure is negative.

Study description	The images of human body and the corresponding data were collected from 3 volunteers. Details listed in Methods. We used the sensing system for human detection by touching human body with our mechanical hand integrated with tactile and olfactory sensing arrays. the data are quantitative.
Research sample	Three volunteers are male or female adult Asians. Their ages are from 21 years old to 40 years old. the sample is representative. We choose people from random age range for human detection.
Sampling strategy	Data acquiring method listed in Supplementary Information and Methods. Each experiment was repeated at least three times independently. The experimental outcomes between independent experiments were in all cases comparable. All data are presented as mean ± standard deviation.
Data collection	Data collection listed in Methods. The sensing arrays were connected to a home-built data serial bus for powering and signal pre-processing. An instrumentation amplifier array (AD 8221) and a data acquisition card (NI 6255) were used to amplify the output voltage signals forty times and collect the amplified signals. A portable resistance detection unit was used to measure the resistive gas responses. Digital force measurement equipment was used to apply external force on the tactile arrays. The sensing arrays were put in an 18 L glass chamber for quantitative gas detection. The gas concentration was controlled by a dynamic gas pumping system. A LabVIEW program was used to gather the data with a sampling frequency of 50000 points per sec from different channels for the next step. All experiment participants were fully voluntary and the construction of all challenging scenarios are under the guidance from the Shanghai fire department.
Timing	The start date of data collecting is 2019/10/10 and the stop date of data collecting is 2021/11/21
Data exclusions	No data were excluded from the analyses.
Non-participation	No participants dropped out participation.
Randomization	Participants were not allocated into experimental groups.

Reporting for specific materials, systems and methods

We require information from authors about some types of materials, experimental systems and methods used in many studies. Here, indicate whether each material, system or method listed is relevant to your study. If you are not sure if a list item applies to your research, read the appropriate section before selecting a response.

Materials & experimental systems

n/a	Involved in the study	
☒	☐	Antibodies
☒	☐	Eukaryotic cell lines
☒	☐	Palaeontology and archaeology
☐	☒	Animals and other organisms
☐	☒	Human research participants
☒	☐	Clinical data
☒	☐	Dual use research of concern

Methods

n/a	Involved in the study	
☒	☐	ChIP-seq
☒	☐	Flow cytometry
☒	☐	MRI-based neuroimaging

Animals and other organisms

Policy information about studies involving animals; ARRIVE guidelines recommended for reporting animal research

Laboratory animals	Balb/c mice (6–8 weeks old, male, Shanghai SLAC Laboratory Animal Co., Ltd, China)
Wild animals	None
Field-collected samples	None

Ethics oversight	Shanghai Institute of Microsystem and Information Technology; Chinese Academy of Sciences; Shanghai Fire Research Institute of MEM; Huashan Hospital of Fudan University

Note that full information on the approval of the study protocol must also be provided in the manuscript.

Human research participants

Policy information about studies involving human research participants

Population characteristics	See above.
Recruitment	Colleagues and students. There are no potential self-selection bias or other biases that may be present and how these are likely to impact results.
Ethics oversight	Shanghai Institute of Microsystem and Information Technology; Chinese Academy of Sciences; Shanghai Fire Research Institute of MEM; All experiment participants were fully voluntary and the construction of all challenging scenarios are under the guidance from the Shanghai fire department.

Note that full information on the approval of the study protocol must also be provided in the manuscript.
